# Human Papillomavirus Is Associated with Breast Cancer in the North Part of Iran

**DOI:** 10.1100/2012/837191

**Published:** 2012-04-01

**Authors:** Afsaneh Sigaroodi, Seyed Alireza Nadji, Farshad Naghshvar, Rakhshandeh Nategh, Habib Emami, Ali Akbar Velayati

**Affiliations:** ^1^Department of Biology, Science and Research Center, Islamic Azad University, Hesarak, Ashrafi Esfahani Highway, Tehran 14778-93855, Iran; ^2^Virology Research Center, NRITLD, Masih Daneshvari Hospital, Shahid Beheshti University of Medical Sciences, Daar-abad, Niavaran, Tehran 19569-44413, Iran; ^3^Department of Pathology, Imam Khomeini Hospital, Mazandaran University of Medical Sciences, Amir-Mazandarani Blvd., Sari 48166-33131, Iran; ^4^Tobacco Prevention and Control Research Center, NRITLD, Masih Daneshvari Hospital, Shahid Beheshti University of Medical Sciences, Daar-abad, Niavaran, Tehran 19569-44413, Iran; ^5^Chronic Respiratory Disease Research Center, NRITLD, Masih Daneshvari Hospital, Shahid Beheshti University of Medical Sciences, Daar-abad, Niavaran, Tehran 19569-44413, Iran

## Abstract

We have analyzed the possible relevance of HPV infection for breast cancer risk among Iranian women from north part of Iran. Among women with breast cancer, 25.9% had positive test results for HPV DNA in breast tumor samples in contrast to 2.4% of women with noncancer status (*P* = 0.002). The infection of HPV has increased the risk of breast cancer (OR 14.247; 95% CI 1.558–130.284, *P* = 0.019). The high-risk HPV genotypes (types 16 and 18) in samples of breast cancer patients were the predominant types (53.34%). Other genotypes detected in breast cancer were HPV-6, HPV-11, HPV-15, HPV-23, and HPV-124, and one isolate could not be genotyped compared to HPV reference sequences. While the sole detected HPV in control specimens was HPV-124. Our study reveals that HPV infection and age are the risk factors in breast cancer development in the north part of Iran.

## 1. Introduction

Breast cancer is the most common cancer in women worldwide, and there were representing 22.9% of all new cancers in 2008 (an estimated 1.378 million new cases) and ranking second overall when both sexes are considered together [1]. Despite of good prognosis of breast cancer, it was also the most common cause of death from cancer, with 13.7% of deaths the world total in woman [1, 2]. Mazandaran province is located in north of Iran and beneath the Caspian Sea. According to the annual report of Health Deputy of Ministry of Health and Medical Education in Iran, breast cancer in women was representing 23.38% of all new cancers in Mazandaran province in 2006-2007 and was the most frequent cancer of women [3].

The incidence of breast cancer is increasing almost everywhere, and this is due in part to increases in risk factors including decreased childbearing and breast feeding, increased exogenous hormone exposure, and harmful dietary and lifestyle changes [2, 4]. On the other hand, breast cancer development might be a consequence of different environmental exposures, including viral infection [4, 5]. Worldwide an estimated 12.1% of all human cancers (about 1.3 million cancer cases) were etiologically related to viral agents in 2002 [5]. Hence, the correlation of viral agents with breast cancer cannot be excluded.

There are controversial reports on the aetiology of HPV in breast cancer around the world. In the last two decades, considerable evidence has been found for a role for HPV in human breast cancer [6–13], but some studies suggested negative relationships [14–16]. In vitro studies have shown that the main oncoproteins E6 and E7 from HPV16 are able to immortalize primary mammary epithelial cells and provided additional evidence for a possible role of this virus in breast carcinogenesis [17–19].

To our knowledge, this is the first study on the presence of HPVs and their relation with in breast cancer in Iranian women. Our previous studies revealed that HPV infection could be considered as a risk factor for the development of lung cancer in Mazandaran population [20, 21]. In the present study, we conducted a case-control study of breast cancer patients and controls and tried to find a viral aetiology by the detection of HPV genome, and to evaluate the possible relevance of this factor for breast cancer risk among north female Iranian population from Mazandaran province.

## 2. Materials and Methods

### 2.1. Tissue Samples

The study protocol was approved by the scientific and ethics committee of the National Research Institute of Tuberculosis and Lung disease, Tehran. A total of 130 blocks of paraffin-embedded tissue including 79 samples diagnosed as breast carcinomas, and 51 noncancer samples as control were retrieved from archive of Imam Khomeini Hospital, Medicine Faculty of Sari city, Mazandaran University of Medical Sciences, Iran between 2002 and 2009. It is worth mentioning that the consents were acquired from the patients during the healthcare and clinical services. Breast fibroadenomas served as control subjects in this study. Furthermore to adjust the environmental confounders, we tried to match the subject's residence place in Mazandaran province between both groups.

### 2.2. DNA Extraction and HPV DNA Detection

Genomic DNAs from tissue sections were prepared according to the methods that previously described [22]. To avoid contamination of the DNA, great care was taken during extraction and PCR (sectioning the blocks to several small groups at different times, using new surgical blade for each sample and filter tips during extraction and PCR).

The adequacy of the DNA in each specimen for PCR amplification was determined by the detection of a 268-base pair (bp) fragment of the *β*-globin gene using the GH20/PC04 primer set [23]. In case of negative results, the PC03/PC04 primer set was used to amplify the 110 bp fragment. For the detection of HPV genome, 3 different primer sets including the GP5+/GP6+ primers [24], the CP primers [25], and the FAP primers [26] were applied in a way that described before [10].

### 2.3. Sequencing and Phylogenetic Analysis

For genotyping of HPV, the positive PCR products were analyzed by sequencing. The DNA sequence was determined with the Big-Dye terminator cycle sequence kit and an ABI 377A sequencer (Applied Biosystems Inc.).

The HPV partial sequences were edited with the BioEdit software program version 7.0.5.2, and then phylogenetic and molecular evolutionary analyses were conducted using MEGA software version 4.0.2 [27]. The UPGMA method was used for phylogenetic reconstructions that were implemented in the MEGA 4.0.2 program. For the UPGMA method, the nucleotide substitution model employed was the Maximum Composite Likelihood. Statistical confidence for the evolutionary trees was assessed by bootstrap (500 replicates). The phylogenetic trees were drawn using the MEGA 4.0.2 program.

### 2.4. Nucleotide Sequence Accession Number

The nucleotide sequences of HPV isolates that determined in this study have been deposited in GenBank data base [accession numbers HM748606–HM748622]. The GenBank accession numbers for the reference HPV nucleotide sequences are as follows:

HPV-1 [V01116], HPV-2 [X55964], HPV-3 [X74462], HPV-4 [X70827], HPV-5 [M17463], HPV-6 [X00203], HPV-7 [X74463], HPV-8 [M12737], HPV-9 [X74464], HPV-10 [X74465], HPV-11 [M14119], HPV-12 [X74466], HPV-13 [X62843], HPV-14 [X74467], HPV-15 [X74468], HPV-16 [K02718], HPV-17 [X74469], HPV-18 [X05015], HPV-19 [X74470], HPV-20 [U31778], HPV-21 [U31779], HPV-22 [U31780], HPV-23 [U31781], HPV-24 [U31782], HPV-25 [X74471], HPV-26 [X74472], HPV-27 [AB211993], HPV-28 [U31783], HPV-29 [U31784], HPV-30 [X74474], HPV-31 [J04353], HPV-32 [X74475], HPV-33 [M12732], HPV-34 [X74476], HPV-36 [U31785], HPV-37 [U31786], HPV-38 [U31787], HPV-39 [M62849], HPV-40 [X74478], HPV-41 [X56147], HPV-42 [M73236], HPV-43 [AJ620205], HPV-44 [U31788], HPV-45 [X74479], HPV-47 [M32305], HPV-48 [U31789], HPV-49 [X74480], HPV-50 [U31790], HPV-51 [M62877], HPV-52 [X74481], HPV-53 [X74482], HPV-54 [U37488], HPV-55 [U31791], HPV-56 [X74483], HPV-57 [X55965], HPV-58 [D90400], HPV-59 [X77858], HPV-60 [U31792], HPV-61 [U31793], HPV-62 [AY395706], HPV-63 [X70828], HPV-64 [U12495], HPV-65 [X70829], HPV-66 [U31794], HPV-67 [D21208], HPV-68 [X67161], HPV-69 [AB027020], HPV-70 [U21941], HPV-71 [AB040456], HPV-72 [X94164], HPV-73 [X94165], HPV-74 [U40822], HPV-75 [Y15173], HPV-76 [Y15174], HPV-80 [Y15176], HPV-81 [AJ620209], HPV-82 [AB027021], HPV-83 [AF151983], HPV-84 [AF293960], HPV-85 [AF131950], HPV-86 [AF349909], HPV-87 [AJ400628], HPV-88 [EF467176], HPV-89 [AF436128], HPV-90 [AY057438], HPV-91 [AF419318], HPV-92 [AF531420], HPV-93 [AY382778], HPV-94 [AJ620211], HPV-95 [AJ620210], HPV-96 [AY382779], HPV-97 [DQ080080], HPV-98 [NC_012744], HPV-99 [NC_012745], HPV-100 [NC_012746], HPV-101 [NC_008189], HPV-102 [DQ080083], HPV-103 [NC_012750], HPV-104 [NC_012750], HPV-105 [NC_012747], HPV-106 [DQ080082], HPV-107 [EF422221], HPV-108 [NC_012213], HPV-109 [NC_012485], HPV-110 [EU410348], HPV-111 [EU410349], HPV-112 [NC_012486], HPV-113 [NC_012748], HPV-114 [NC_013931], HPV-115 [NC_013591], HPV-116 [NC_013035], HPV-117 [GQ246950], HPV-119 [GQ845441], HPV-120 [GQ845442], HPV-121 [NC_014185], HPV-122 [GQ845444], HPV-123 [GQ845445], HPV-124 [GQ845446], PcPV [X62844], RhPV1 [M60184].

### 2.5. Statistical Data Processing

Data were processed by SPSS statistical software program version 16.0. The correlations were subjected to *χ* ^2^ (Pearson chi-square) and Fisher's exact test. Odds ratios and logistic regression were also calculated. Statistical significance was set as a *P*-value less than 0.05.

## 3. Results

The characteristics of study subjects including age, HPV DNA, HPV genotype, and breast cancer histopathologic types are shown in Table 1. A total of 130 individuals, including 79 breast cancer patients and 51 noncancer controls, were recruited into this study (Table 1).

The mean ages were 47.77 ± 12.552 (S.D.) and 34.20 ± 9.704 (S.D.) years in breast cancer and control groups, respectively (*P* ≤ 0.0001, Table 1). The most abundant type of breast cancer determined histologically was IDC (84.8%), followed by ILC (10.1%), MC (2.5%), DC, and IDL-ILC (1.3%) (Table 1).

Statistical difference was observed in the HPV DNA status (25.9% versus 2.4 0%, *P* = 0.002) and HPV genotype (caser risk type, *P* = 0.005; tissue tropism type, *P* = 0.002) between these two groups (Table 1). HPV infection has increased the risk of breast cancer and had an OR of 13.953 (95% CI 1.762–110.526; *P* = 0.002, Table 1).

**Figure 1:**
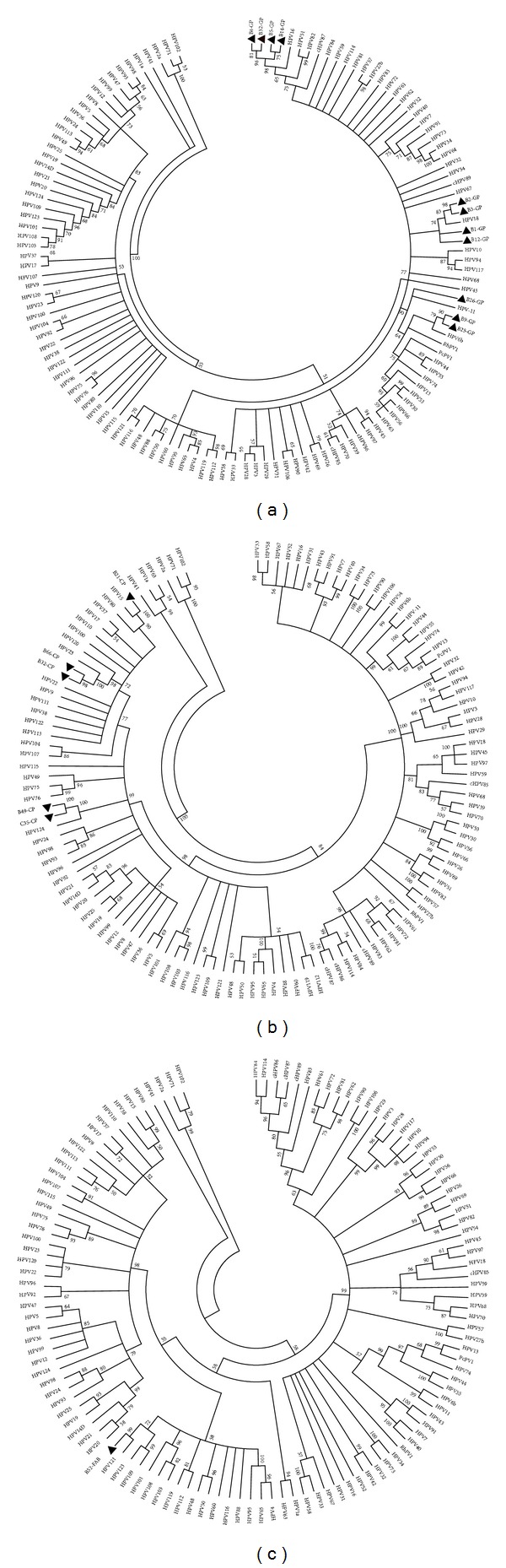
Phylogenetic analysis of L1 gene partial sequences amplified by (a) GP5+/GP6+, (b) CP primer set, and (c) FAB primer set in HPV isolates. The trees were constructed by the UPGMA method using MEGA 4.0.2. The HPV isolates are marked with black triangles.

The high-risk HPV types (HPV 16 and 18) were more prevalent than other HPV types in the cases (14.0% versus 10.5%, Table 1). The HPV genotypes in samples of breast cancer patients were 26.67% for HPV-16 (4 isolates) and HPV-18 (4 isolates), 13.3% for HPV-23 (2 isolates) and HPV-6 (2 isolates), 6.67% for HPV-11 (1 isolate), HPV-15 (1 isolate), and HPV-124 (1 isolate), and one isolate could not be genotyped compared to HPV reference sequences (Table 1) while the sole detected HPV in control specimens was HPV-124 (Table 1). The constructed phylogenetic trees of the HPV isolates are shown in Figure 1 in 3 separate parts. The co-infection with different HPV types (HPV-16 and HPV-23) was observed only in one sample, B32, which was detected by GP and CP primer sets (Figures 1(a) and 1(b)).

In Table 2, HPV DNA detection rate is stratified by age and breast cancer histopathologic types. No significant difference was observed among breast tumor types considering HPV status (Table 2). Due to incomparability of the age means of case and control and to avoid the effect of the age parameter, a logistic regression model was run. Data shown that age (OR 0.873, 95% CI 0.822–0.927; *P* ≤ 0.0001) and HPV infection (OR 14.247, 95% CI 1.558–130.284; *P* = 0.019) are the significant risk factors in breast carcinogenesis in the studied women in north part of Iran, Mazandaran province (Table 3).

## 4. Discussion

HPVs belong to Papillomaviridae family, and epidemiological studies have shown that a persistent HPV infection is the most important risk factor for cervical cancer [28, 29]. HPVs are also considered to be one of the risk factors for human breast carcinogenesis. The concept of the relationship between HPV and breast cancer is based on the identification of HPV genome sequence in breast cancer tissues and immortalization of primary mammary epithelial cells by high-risk HPV [17–19]. However, involvement of HPV in breast cancer is controversial. Since 1992, a growing number of studies had identified HPVs in breast tumors by PCR around the world, with a positivity variation from 4% to 86% for suggesting negative [14–16, 30, 31] or positive relationships [6–13, 32–36]. These results reflect the controversy in the role of HPV in the pathogenesis of breast cancer. The controversy is influenced by the technical limitations and the epidemiology of HPV in different geographical area [36–38].

In our previous studies [20, 21], we showed that HPV infection may be associated with the development of lung cancer in Mazandaran, north part of Iran. It was worth mentioning that the HPV infection was the risk factor only in male gender, not in females [20, 21]. In this study, we have evaluated the association between the breast cancer and HPV infection in the north part of Iran. For the detection of HPV DNA, FFPE tissues were analyzed by PCR using 3 different primer sets. As shown in Table 1, the detection frequency of HPV DNA in breast cancer patients was significantly higher than that of control patients (25.9% versus 2.40%, *P* = 0.002). We suggest that HPV infection is significantly related to breast cancer in women live in Mazandaran province (OR 14.247, 95% CI 1.558–130.284; *P* = 0.019, Table 3). Regarding to HPV prevalence in breast cancer, a meta-analysis study that explores the correlation between HPV infection and risk of breast carcinoma revealed that HPV prevalence was lowest in Europe (12.91%) and highest in Oceania (42.11%) followed by Asia (32.42%) [38].

High-risk HPV types, such as HPV-16 and HPV-18, were detected in our study, and they comprised the majority of isolates (Table 1). Although there are great variability in the HPV detection rate worldwide, the majority of HPV types that are detected are the oncogenic types, HPV-16 and -18 [6–8, 11, 33, 38]. Our results are in agreement with these previous reports (Table 1). In addition, we detected cutaneous HPV types (HPV-15, -23, and -124). The presence of cutaneous HPV types was reported in the previous studies among women with breast cancer or at increased risk for breast cancer [10, 39]. The only isolate that screened by the FAB primer set has no significant similarity to any known HPV types. Pairwise distance calculation revealed that the HPV type 121 is the most similar type to the isolate (Figure 1(c)) and the distance estimation is 0.235.

## 5. Conclusions

The pathogenesis of breast cancer is complex. This study demonstrates the presence of HPV genome in tumor tissues in women with breast cancer in north part of Iran. HPV infection is associated with the development of breast cancer in women live in Mazandaran province. Further studies are needed to clarify the role and the risk assessment of HPV in human breast cancer. Confirming an etiologic role for HPV in breast cancer in Iranian females may help develop vaccine strategies for combating this increasingly common cancer.

## Figures and Tables

**Table 1:** The characteristics of study subjects and prevalence of HPV DNA status in breast cancer patients and noncancer controls.

Parameter	Cases^a^ (*N* = 79)	Controls^a^ (*N* = 51)	*P*-value
Age (year ± SD)	47.77 ± 12.552	34.20 ± 9.704	**≤0.0001 **(*t*-test)

HPV			0.002
Positive	15 (25.9)^b^	1 (2.4)	**O** **R** 13.953
Negative	43 (74.1)	40 (97.6)	(95% CI 1.762–110.526)

HPV genotype (% within type)			
HPV-124	1 (6.25)	1 (100)	
HPV-23	2 (12.5)		
HPV-18	4 (25)		
HPV-16	4 (25)		
HPV-15	1 (6.25)		
HPV-11	1 (6.25)		
HPV-6	2 (12.5)		
Unknown	1 (6.25)		

HPV genotype (% within subjects)			
High-risk type	8 (14)	0	**0.005 **
Low-risk type	6 (10.5)	1 (2.4)	
Negative	43 (75.5)	40 (97.6)	

HPV genotype (% within subjects)			
Mucosal type	11 (19)	0	**0.002**
EV-cutaneous type	4 (6.9)	1 (2.4)	
Negative	43 (74.1)	40 (97.6)	

Tumor type (% within tumor type)			
IDC	67 (84.8)		
ILC	8 (10.1)		
IDC-ILC mix	1 (1.3)		
MC	2 (2.5)		
DC	1 (1.3)		

^a^Some of the subjects have been considered as missing value after quality examination of nucleic acid extraction.

^b^Numbers in parentheses are percentages.

**Table 2:** HPV status according to age groups and breast cancer histopathologic types.

Variable	Cases		Controls		*P*-*value *
	HPV positive (%)	Number of subjects	HPV positive (%)	Number of subjects	
Age (year)					
<35	3 (49.9)	7	0	22	0.01
≥35	12 (23.5)	51	1 (7.7)	19	0.097

*P*-*value *	0.360		0.463		

Tumor type					
IDC	11 (22.9)	48			
ILC	4 (66.7)	6			
IDC-ILC	0	1			
MC	0	2			
DC	0	1			

*P*-*value *	0.144				

**Table 3:** The Breast cancer risk estimation using the logistic regression model according to HPV status and Age in Iran, Mazandaran province.

Variable	Number	OR	CI 95%	*P*-value
Age	99	0.873	0.822–0.927	0.000
HPV	130	14.247	1.558–130.284	0.019

## Abbreviations

HPV: Human papillomavirus
PCR: Polymerase chain reaction
FFPE: Formalin-fixed paraffin-embedded
bp: Base pair
SD: Standard deviation
OR: Odds ratio
CI: Confidence interval
IDC: Invasive ductal carcinoma
ILC: Invasive lubular carcinoma
MC: Medullary carcinoma
DC: Ductal carcinoma in situ.
